# I-Motif Structures Formed in the Human c-MYC Promoter Are Highly Dynamic–Insights into Sequence Redundancy and I-Motif Stability

**DOI:** 10.1371/journal.pone.0011647

**Published:** 2010-07-19

**Authors:** Jixun Dai, Emmanuel Hatzakis, Laurence H. Hurley, Danzhou Yang

**Affiliations:** 1 College of Pharmacy, The University of Arizona, Tucson, Arizona, United States of America; 2 BIO5 Institute, The University of Arizona, Tucson, Arizona, United States of America; 3 Arizona Cancer Center, The University of Arizona, Tucson, Arizona, United States of America; 4 Department of Chemistry, The University of Arizona, Tucson, Arizona, United States of America; German Cancer Research Center, Germany

## Abstract

The GC-rich nuclease hypersensitivity element III_1_ (NHE III_1_) of the c-MYC promoter largely controls the transcriptional activity of the c-MYC oncogene. The C-rich strand in this region can form I-motif DNA secondary structures. We determined the folding pattern of the major I-motif formed in the NHE III_1_, which can be formed at near-neutral pH. While we find that the I-motif formed in the four 3′ consecutive runs of cytosines appears to be the most favored, our results demonstrate that the C-rich strand of the c-MYC NHE III_1_ exhibits a high degree of dynamic equilibration. Using a trisubstituted oligomer of this region, we determined the formation of two equilibrating loop isomers, one of which contains a flipped-out cytosine. Our results indicate that the intercalative cytosine^+^–cytosine base pairs are not always necessary for an intramolecular I-motif. The dynamic character of the c-MYC I-motif is intrinsic to the NHE III_1_ sequence and appears to provide stability to the c-MYC I-motif.

## Introduction

c-MYC is a potent oncogene whose protein product is a transcription factor that controls many genes associated with cell growth and cell fate determination [1], [2], [3]. Overexpression of the c-MYC proto-oncogene is associated with many human malignancies, including colon, breast, prostate, cervical, and lung carcinomas, osteosarcomas, lymphomas, and leukemias [4], [5], [6], [7], [8], [9], [10], [11], [12]. In addition, elevated levels of c-MYC expression are often associated with poor therapeutic prognosis. c-MYC overexpression can be caused by different mechanisms, including gene amplification [13], [14], translocation [15], [16], [17], and simple upregulation of transcription [1], [4]. The transcriptional regulation of c-MYC expression is complex and involves multiple promoters and transcriptional start sites, with P1 and P2 being the predominant promoters [18]. A highly conserved NHE III_1_, a 27-base-pair sequence located –142 to –115 base pairs upstream from the P1 promoter, has been shown to be required for 80–95% of c-MYC transcription, regardless of whether the P1 or P2 promoter is used [19]. The NHE III_1_ element has been shown to form transcriptionally active and silenced forms in the promoter [20], [21]. The polyguanine/polycytosine NHE III_1_ element can form DNA secondary structures, namely G-quadruplex and I-motif [22], [23], whose *in vivo* formation may be induced by transcription-generated superhelicity [24], [25], [26], [27]. The formation of G-quadruplex has been shown to be critical for c-MYC transcriptional silencing [28], [29], [30], [31], and compounds that stabilize the G-quadruplex repress c-MYC gene expression [28], [32]. The folding topology [33], [34] and molecular structure [35] of the major c-MYC G-quadruplex, which is formed by the four 3′ consecutive runs (2345) of guanines, have been determined by us and others.

**Figure 1:**
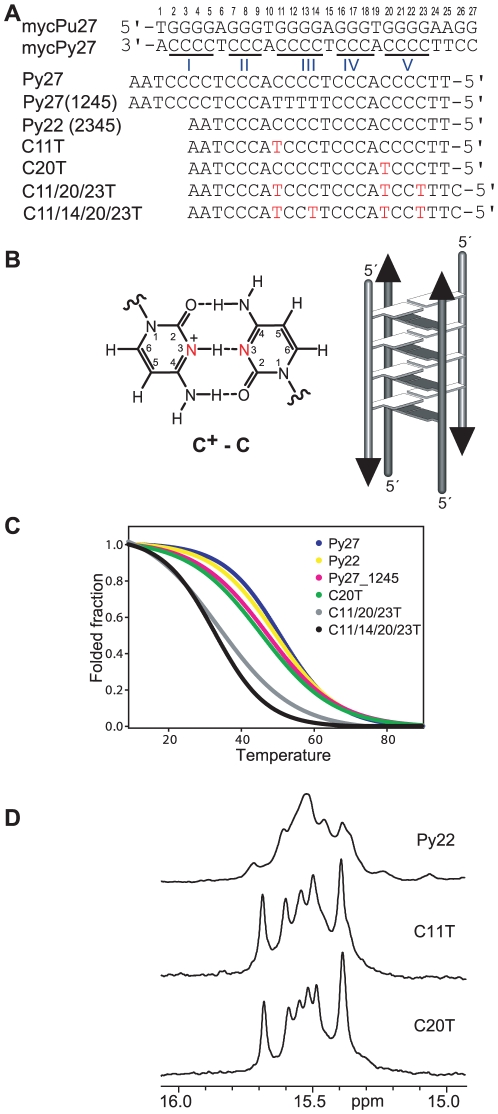
The c-MYC NHE III_1_ sequences and I-motif structure. (A) The promoter sequence of the NHE III_1_ element of the c-MYC gene and its modifications. mycPu27 is the wild-type 27-mer G-rich sequence of the c-MYC NHE III_1_; Pu22 is the modified G-rich sequence that adopts the single predominant c-MYC promoter G-quadruplex and was used for structure determination [35]; mycPy27 is the wild-type 27-mer C-rich sequence of the c-MYC NHE III_1_; Py27 is the wild-type C-rich promoter sequence with a 3′-AA; Py27(1245) is the modified Py27 that can only form the (1245) form of the c-MYC I-motif; Py22 is the truncated wild-type Py27 with a 3′-AA that can only form the (2345) form of the c-MYC I-motif; C11T, C20T, C11/20/23T, and C11/14/20/23T are the modified Py22 with single C-to-T substitutions. The numbering system is based on the G-rich strand and is shown above mycPy27. (B) A C^+^-C base pair (left), and a schematic drawing of a four-stranded I-motif structure (right). (C) CD melting curves of various C-rich c-MYC promoter sequences shown in (A) at pH 5.5. (D) Imino regions of 1D ^1^H NMR spectra of Py22, C11T, and C20T at 25°C, pH 5.5.

The C-rich strand of the NHE III_1_ sequence (mycPy27, Figure 1A) can adopt another DNA secondary structure, the I-motif. An I-motif is a four-stranded structure consisting of parallel-stranded duplexes zipped together in an antiparallel orientation by intercalated, hemiprotonated cytosine^+^–cytosine (C^+^-C) base pairs [36], [37], [38], [39], [40], [41] (Figure 1B). It has been observed that the I-motif formed in the c-MYC promoter also interacts with small molecule compounds that control gene transcription (unpublished data). The 27-mer mycPy27 (Figure 1A) contains five runs of cytosines and can form multiple I-motif structures. It has been previously suggested that the major I-motif formed in this sequence is the (1245) form, utilizing the I/II and IV/V C-runs of the c-MYC NHE III_1_ (Figure 1A) [23]. However, in this study we found that the II, III, IV and V C-runs in the (2345) tract formed an I-motif which was more stable than the (1245) I-motif (Figure 1A). The major c-MYC I-motif appears to be formed at near-neutral pH. We have determined the folding structure of this major I-motif formed in the c-MYC promoter using NMR spectroscopy and mutational analysis. While this study represents the first well-defined folding structure of DNA I-motifs formed in a wild-type promoter sequence of human proto-oncogenes, our results show that the C-rich strand of the c-MYC NHE III_1_ exhibits a high degree of sequence redundancy and dynamic equilibration. This dynamic character is intrinsic to the c-MYC NHE III_1_ sequence and appears provides stability to the c-MYC I-motif. In addition, our results indicate that, surprisingly, the intercalative C^+^-C base pairs are not always necessary in an intramolecular I-motif.

## Results

### The (2345) form is the major I-motif structure formed in the C-rich strand of the c-MYC NHE III_1_ sequence

Using mutational analysis, we first tested the stability of the wild-type mycPy27, mutated Py27(1245) that can only adopt the (1245) form, and the truncated Py22 that can only adopt the (2345) form (Figure 1A) using both CD and NMR spectroscopy. We found that, at pH 5.5, the wild-type mycPy27 has a melting temperature around 51°C, Py22 has a melting temperature of 49.5°C, and Py27(1245) has a melting temperature of 47.5°C at pH 5.5, respectively, as determined by CD. (Figure 1C). This result indicates that the (2345) form is more favored over the (1245) form and is likely to be the major form, just as in the G-rich strand.

**Figure 2:**
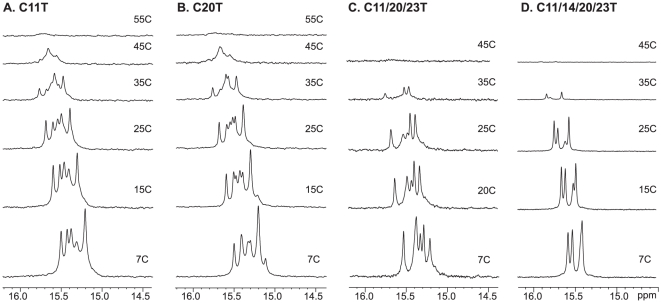
Variable temperature 1D ^1^H NMR of c-MYC I-motif sequences. Imino proton regions of variable temperature 1D ^1^H NMR spectra of C11T, C20T, C11/20/23T, and C11/14/20/23T at pH 5.5.

We then examined the (2345) region of the c-MYC promoter C-rich sequence (Figure 1A), Py22, which forms the major c-MYC I-motif. The C-rich sequence has a much higher degree of sequence redundancy than the G-rich sequence, because not all cytosines can be used simultaneously in C^+^-C base-pair formation (Figure 1B). The one-dimensional ^1^H NMR spectrum of the wild-type sequence Py22 (Figure 1A) is shown in Figure 1D (upper). The imino protons between 15–16 ppm are characteristic of an I-motif structure [42]. The distinct chemical shifts around 15–16 ppm for the I-motif cytosine imino protons result from the downfield shifting by the combination of hydrogen-bonding and positively charged protonation site. The relatively strong signals at 15–16 ppm shown in Figure 1D indicate the stable formation of I-motif structures. However, the broad envelopes in 1D ^1^H NMR indicate the presence of a dynamic equilibrium of multiple conformers. Based on our experience and insight obtained from the G-quadruplex structures formed in the G-rich strand, we have systematically tested the mutated sequences with single C-to-T mutations at positions 11, 13, 14, 20, and 23. We have also found that a 3′-AA sequence can stabilize the c-MYC I-motif. The Py22 sequences with a single C-to-T mutation at position 11 or 20, respectively, give rise to good NMR spectral properties and were used for the NMR analysis (Figure 1D). These two sequences are named C11T and C20T, respectively. The sharp and well-resolved NMR spectral lines of imino protons located between 15 and 16 ppm indicate the formation of stable and well-defined I-motif structures. The melting temperatures of C11T and C20T are around 45.5°C at pH 5.5 (Figure 1C, Figures 2A and 2B). The I-motif structures formed by C11T and C20T appear to be of a unimolecular nature, based on CD and NMR variable temperature studies which showed a concentration-independent melting temperature.

### The C11T and C20T sequences form similarly folded I-motif structures

**Figure 3:**
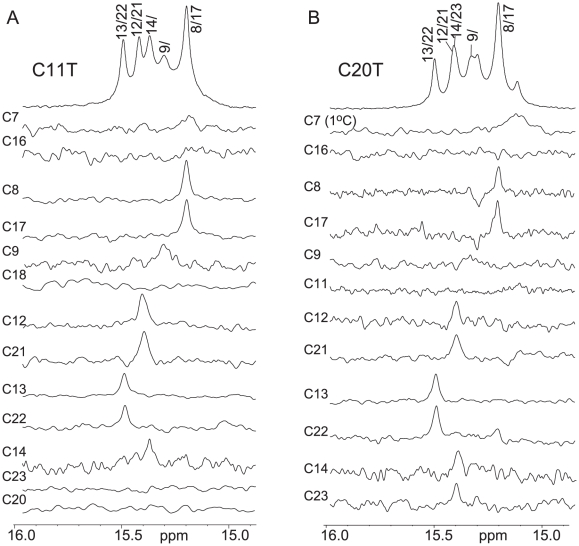
Imino proton assignments of C11T and C20T c-MYC I-motif. Imino proton assignments of C11T (A) and C20T (B) using 1D ^15^N-filtered experiments on site-specific 6% ^15^N-labeled oligonucleotides. Each site-specifically labeled cytosine is shown above its corresponding spectrum. The assignment of all cytosine imino protons is shown above the 1D spectra of the corresponding sequence. All samples are prepared at pH 5.5. NMR experiments were performed at 7°C except for the C7-labeled C20T which was performed at 1°C.

**Figure 4:**
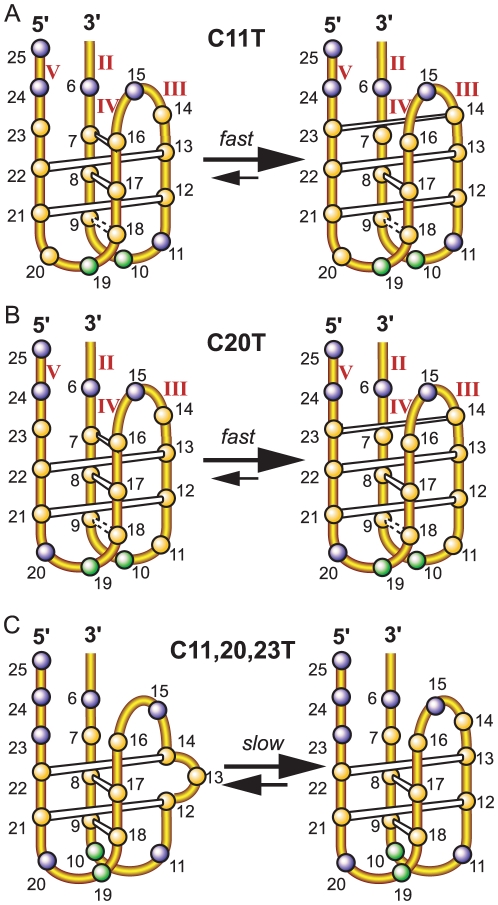
Folding structures of the c-MYC I-motifs. Schematic drawing of the folding structures of the c-MYC I-motifs formed in the C11T sequence (A), the C20T sequence (B), and the two equilibrating conformations in the C11/20/23T sequence (C). The C^+^-C base pairs are shown in white boxes. The dashed boxes indicate the possible C^+^-C base pairs that are in dynamic equilibrium and thus show weaker and broader C^+^-C imino peaks. (cytosine  =  yellow sphere, adenine  =  green sphere, thymine  =  blue sphere.)

Using site-specific low-concentration (6%) incorporation of a uniformly ^15^N-labeled cytosine nucleoside at each cytosine position of the C11T and C20T sequences (Figure 1A) one at a time, the imino protons of cytosine residues involved in the C^+^-C base pairs can be directly determined by NMR [43]. Since the two cytosines forming a C^+^-C base pair share one imino H3 hydrogen, the imino proton in a C^+^-C base pair has a one-bond coupling to the N3 atoms of both cytosines and hence can be unambiguously assigned by the 1D ^15^N-filtered HMQC experiment. The assignment of each cytosine imino proton involved in the hemiprotonated C^+^-C base-pairs of C11T is shown in Figure 3A, which enabled us to identify each partner C^+^-C base pair involved in the I-motif structure(s) (Figure 4A). For example, the site-specific substitution of a ^15^N-uniformly labeled cytosine at the C21 and C12 positions gives rise to an HMQC peak with the same cytosine imino proton at 15.4 ppm, indicating that C21 and C12 form a C^+^-C base pair (Figure 3A). Based on this method, three C^+^-C base pairs are clearly detected between C22-C13, C17-C8, and C21-C12, respectively, as each pair shares the same cytosine imino proton in the ^15^N-filtered HMQC data. In addition, a weak C^+^-C imino peak was also detected for the C14-labeled C11T sequence.

We have also carried out 1D ^15^N-filtered HMQC experiments for the site-specifically labeled C20T sequence. The assignment of each cytosine imino proton involved in the C^+^-C base pairs of C20T is shown in Figure 3B. The same three C^+^-C base pairs observed for the C11T sequence, namely, C22-C13, C17-C8, and C21-C12, were also clearly detected in the 1D ^15^N-filtered HMQC spectra of C20T. In addition, a C^+^-C base pair was detected between C23-C14 in C20T, while the intensity of the imino proton of this C23-C14 base pair appears to be weaker than those of the other three base pairs.

Based on the NMR data, the I-motif structures formed by the C11T and C20T sequences can be determined. The two I-motifs have similar folding structures with three lateral loops (Figures 4A and 4B). Both I-motif structures contain the same three C^+^-C base pairs, i.e., C22-C13, C17-C8, and C21-C12. C20T contains a clearly detectable, albeit weaker, C^+^-C base pair between C14 and C23. The same C^+^-C imino proton can be clearly detected for C14 in the C11T sequence as well. Thus the C23-C14 base pair appears to also form in C11T. In addition, a weaker and broader C^+^-C imino peak was detected for the C9-labeled C11T and C20T, and to a lesser extent, for the C7-labeled C11T and C20T (only at 1°C). Thus C9 and C7 appear to be involved in C^+^-C base pairing, likely with C18 and C16, respectively. However, the C18-C9 and C16-C7 base pairs appear to be more mobile and dynamic. The I-motif structures formed by the C11T and C20T sequences both contain three lateral loops. The bottom two lateral loops are 2-nt long (Figures 4A and 4B). Interestingly, for the top lateral loop, C14 and C16 are located in the same loop region. The lateral loops of a stable I-motif structure all contain at least two bases, as shown in the available structural data [37], [38], [40], [41], thus C23-C14 and C16-C7 are unlikely to form at the same time. The C16-C7 base pair is right above the existing C22-C13 base pair and was originally expected to form instead of the C23-C14 base pair. However, the C23-C14 base pair can be detected much more clearly than C16-C7 in both the C11T and C20T sequences (Figure 3), indicating that the C23-C14 base pair is more stable than the C16-C7 base pair and that the C23-C14 base pair is formed in the majority of the populations of the two I-motifs (Figures 4A and 4B, right), whereas the C16-C7 base pair is only formed transiently in the minor populations (Figures 4A and 4B, left).

Notably, no C^+^-C imino peak was detectable for the C11-labeled C20T or the C20-labeled C11T even at 1°C, indicating that C11 and C20 are in the loop region and hence are not involved in a C^+^-C base pairing. We have also prepared and tested a Py22 sequence with dual C-to-T substitutions at positions 11 and 20, C11/20T, which exhibits a NMR spectrum, and a melting temperature, very similar to those of C11T and C20T (data not shown).

### Two stable I-motifs in equilibrium are formed in the C11/20/23T sequence

It is interesting to note that the C23-C14 base pair is more stably formed than the C16-C7 base pairs in both the C11T and C20T sequences (Figure 3). For the C23-C14 base pair, C23 was shown to be less detectable than its base-paired partner C14 (Figure 3A), indicating that it is more dynamic in nature. Therefore, we have carried out further mutational studies to examine the effect of the C-to-T substitution at positions 23 and whether this mutation could induce the stable formation of the C7-C16 base pair. The tri-substituted Py22 sequence, C11/20/23T (Figure 1A), exhibits better NMR spectral resolution at low temperatures (Figure 2C). The melting temperature of C11/20/23T is around 34°C (Figure 1C), significantly lower than those of C11T and C20T, and is also concentration-independent as shown by CD and NMR.

**Figure 5:**
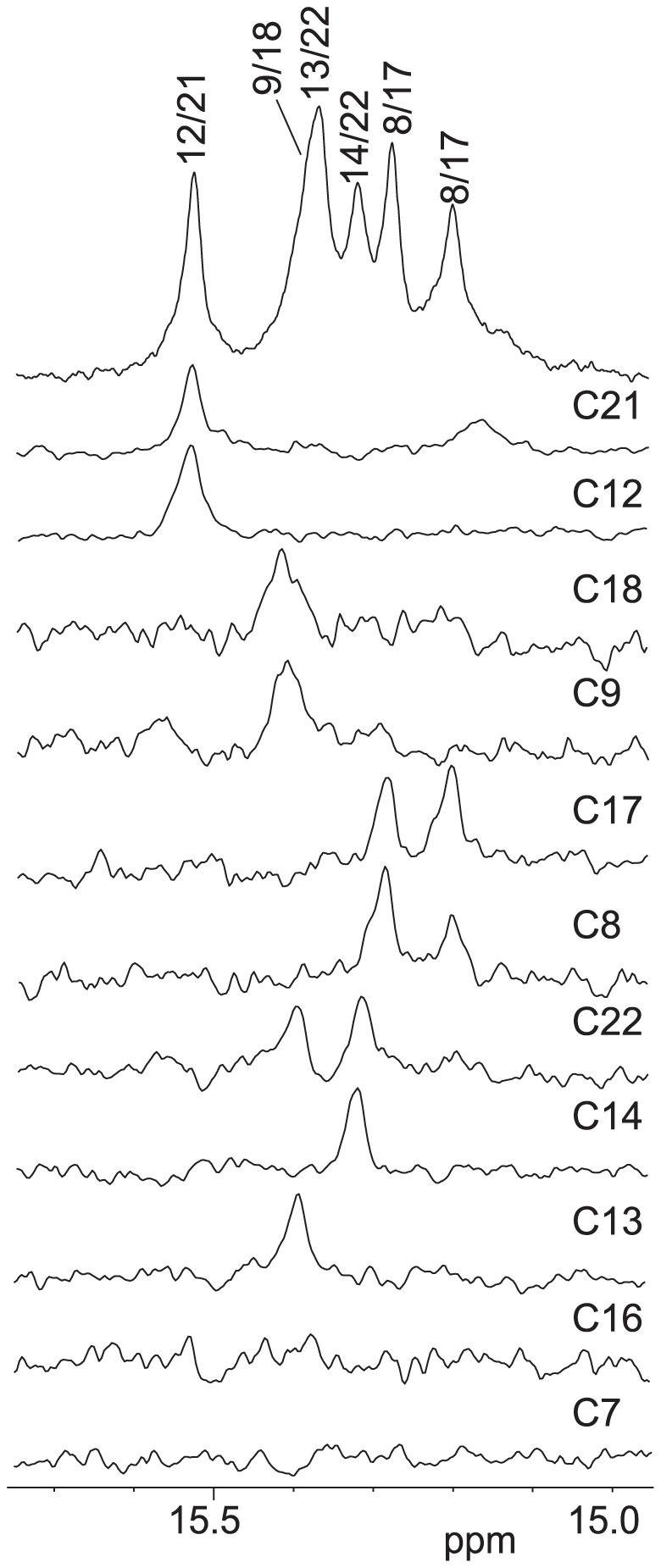
Imino proton assignment of C11/20/23T c-MYC I-motif. Imino proton assignments of C11/20/23T using 1D ^15^N-filtered experiments on site-specific ^15^N-labeled oligonucleotides. Each site-specifically labeled cytosine is shown above its corresponding spectrum. The assignment of all cytosine imino protons is shown above the 1D ^1^H spectrum of C11/20/23T. All samples are at pH 5.5. NMR experiments were performed at 7°C.

Using the same method described previously, we have site-specifically labeled the C11/20/23T sequence by ^15^N-cytosine nucleoside at each cytosine position one at a time and carried out 1D ^15^N-filtered HMQC experiments for each oligonucleotide molecule. The assignment of each cytosine imino proton involved in the C^+^-C base pairs of C11/20/23T is shown in Figure 5. In addition to the C21-C12 base pair, we are able to clearly detect the C18-C9 base pair. However, the C16-C7 base pair still does not appear to form (Figure 5). Surprisingly, each of the C8- and C17-labeled C11/20/23T DNA samples shows two sets of imino peaks at the same time (Figure 5). In addition, the C22-labeled DNA shows two imino peaks, with each corresponding to the single imino peak arising from the C13- and C14-labeled C11/20/23T sample. This result indicates that C22 is base-paired with C13 in one conformation and with C14 in another, and that C8 and C17 are base-paired and the C17-C8 base pair is involved in two different conformations. This unexpected result indicates that the C11/20/23T sequence forms two stable I-motif conformations, as shown in Figure 4C. The two conformations are in slow equilibrium on the NMR time scale (ms), since they have sharp and well-resolved NMR peaks. Both I-motif structures contain four C^+^-C base pairs, three of which are the same (Figure 4C). In the isomer shown in Figure 4C right, C22 is base-paired with C13, while in the isomer shown in Figure 4C left, C22 is base-paired with C14, with C13 looped out. Thus C22 gives rise to two distinct imino peaks with base-pairing to either C13 or C14. These two conformations also affect the neighboring C17-C8 base pair which gives rise to two different imino peaks in the two conformations. Both isomers have three lateral loops. The right isomer contains two 2-nt loops at the bottom and one 3-nt loop at the top, while the left isomer also contains the same two 2-nt loops at the bottom but only a 2-nt loop at the top (Figure 4C), which may explain why the C16-C7 base pair is not able to form in the C11/20/23T sequence.

It is interesting to note that in the second conformation (Figure 4C left), the C13 residue needs to be looped out. We prepared two modified Py23 sequences with one additional C-to-T mutation at either position 13 or 14 to isolate each isomer. The C11/14/20/23T mutant gives rise to an NMR spectrum of excellent spectral quality (Figure 2D), with four cytosine imino peaks for the four C^+^-C base pairs, indicating the stable I-motif formation of the folding pattern shown in Figure 4C right. In contrast, the C11/13/20/23T mutant gives rise to an NMR spectrum with much weaker imino resonances, indicating an unstable I-motif formation without the equilibrating conformation. This has also been confirmed by molecular modeling studies, which showed that the isomer shown in Figure 4C left was not stable by itself (data not shown). It is thus suggested that the isomer in Figure 4C left can only be stably formed with the presence of the isomer in Figure 4C right. The melting temperature of C11/14/20/23T is around 33°C, very close to that of C11/20/23T (34°C).

### The (2345) form I-motif structures of the c-MYC promoter can be stably formed at near neutral pH

**Figure 6:**
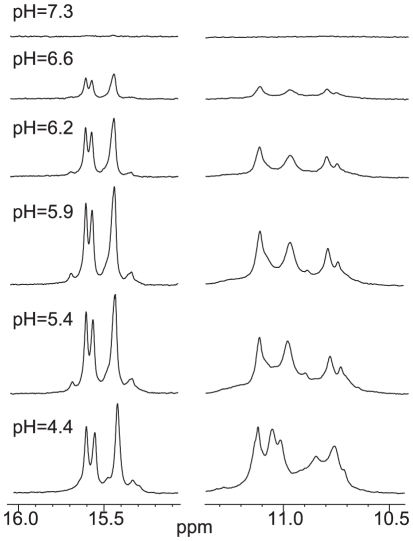
Variable pH 1D ^1^H NMR of C11/14/20/23T c-MYC I-motif. Cytosine- and thymine- imino regions of 1D ^1^H NMR spectra of C11/14/20/23T at various pHs at 7°C.

We have examined by NMR the formation and stability of the I-motif structures in the four variant c-MYC C-rich sequences, i.e., C11T, C20T, C11/20/23T, and C11/14/20/23T, at various pHs. Significantly, the stable formation of I-motif structures in these sequences can be clearly seen at pH 6.6. The 1D ^1^H NMR spectra of C11/14/20/23T at various pHs is shown in Figure 6. Even for this C11/14/20/23T sequence which appears to be the least stable among the four variant c-MYC C-rich sequences (Figure 1A) as indicated by the lowest melting temperature, the stable formation of a I-motif structure was clearly detected at pH 6.6.

## Discussion

### Major I-motif formed in mycPy27

The 27-mer C-rich strand in the c-MYC NHE III_1_ (mycPy27, Figure 1A) contains five runs of cytosines and can form multiple I-motif structures. We found that the (2345) I-motif formed in Py22 is more stable than the (1245) I-motif formed in Py27(1245) and appears to be the major I-motif formed in the C-rich strand in the NHE III_1_ of the c-MYC promoter. The Py22 sequences with a single C-to-T mutation at positions 11 (C11T) or 20 (C20T), respectively, gave rise to well-resolved NMR spectra and were used for the NMR analysis. The C11T and C20T sequences appear to form the same major I-motif as in the wild-type Py22 sequence. The C11 in the C20T sequence and the C20 in the C11T sequence, respectively, are shown to be in the loop region and not involved in any C^+^-C base pairing.

### The lateral loops of a stable I-motif structure must each contain at least two bases

We have determined the folding patterns of the I-motif formed in C11T and C20T using NMR and selective incorporation of ^15^N-labeled cytosine nucleosides at each cytosine. The folding structures of the two I-motifs are essentially the same (Figures 4A and 4B), being the chair-type I-motif consisting of five C^+^-C base pairs: C22-C13, C17-C8, C21-C12, C23-C14, and a more dynamic C18-C9 base pair. The C16-C7 base pair may be transiently formed; however, as C14 and C16 are located in the same lateral loop region, the C23-C14 and C16-C7 base pairs are unlikely to form at the same time as the concurrent formation of both base pairs would make the top lateral loop 1 nt long. Thus it is indicated that the lateral loops of a stable I-motif structure must each contain at least two bases. This is also in agreement with the available structural data [37], [38], [40], [41]. The major (2345) form I-motif formed in mycPy27 thus has a loop size of 2 nt for all three lateral loops, in contrast to the loop arrangement of 2 nt, 6 nt, and 2 nt for the (1245) form that was previous reported [23].

### Unexpected formation of non-intercalative adjacent C^+^-C base pairs and its indication for dynamic equilibrium

For the major (2345) form I-motif structure formed in mycPy27, the intercalative C16-C7 base pair from the C-tract duplex pair (II/IV) would be right above the existing C22-C13 base pair from the III/V C-tract duplex (Figures 4A and 4B, left). Thus the C16-C7 base pair was originally expected to form in the (2345) form with three 2 nt loops. However, unexpectedly, the C23-C14 base pair appears to be more stably formed than the C16-C7 base pair in both the C11T and C20T I-motif (Figure 3). Thus it is indicated that, in an intramolecular I-motif structure, a parallel C^+^-C base pair (i.e., C23-C14) may be more preferred than the intercalative one (i.e., C16-C7) for the terminal C^+^-C base pairs (Figures 4A and 4B, right). This may be explained from the structure point of view, as the stacking interaction between the intercalative C^+^-C base pairs is not extensive. The non-intercalative (parallel) C^+^-C base pairs with greater internal spacing may be favored at the more flexible ends due to the presence of the positive charges on the C^+^-C base pairs. Additionally, it was observed that the C^+^-C base pairs are more readily formed between the 5′ C-tract and its partner, i.e., III/V, as compared to the 3′ C-tract and its partner (II/IV); e.g., the C23-C14 base pair (III/V) is much more stably formed than C16-C7 (II/IV), and the C18-C9 base pair (II/IV) appears to be quite dynamic. Whether this is true for other intramolecular I-motif-forming C-rich sequences needs to be further tested.

Notably, although the C16-C7 base pair appears to be more mobile, it can still be detected in both the C11T and C20T sequences. As the formation of the C23-C14 and C16-C7 base pairs are exclusive of each other, the I-motif structures formed by the C11T and C20T sequences thus appear to be in a dynamic equilibrium of two conformers: the C23-C14 base pair is formed in the major conformer, while the C16-C7 base pair is formed in the minor conformer (Figures 4A and 4B). The two conformers appear to be in fast exchange mode on the NMR time scale.

### C11/20/23T forms two equilibrating I-motif isomers, one of which contains an unexpected flipped-out cytosine

As C23 is found to be more mobile than its partner C14 in the unexpectedly formed base pair C23-C14; we tested the effect of an additional C-to-T mutation at position 23 using the C11/20/23T sequence (Figure 1A). To our surprise, however, the elimination of the C23-C14 base pair did not stabilize the C16-C7 base pair (Figure 5). Instead, the mutation of C23 results in the formation of two equilibrating isomers (Figure 4C). In addition to the C22-C13 base pair that was observed in the parent I-motif (Figure 4C right), the C14 residue was also shown to pair with C22 in a second conformer, in which C13 is looped out (Figure 4C left). The two equilibrating isomers appear to be in slow exchange on the NMR time scale. Although this C13-flipped out loop isomer is not stable by itself, its formation appears to preclude the formation of the C16-C7 base pair, as the lateral loops of a stable i-motif structure must contain at least two bases.

### Dynamic characters of the I-motif formed in the c-Myc NHE III_1_

We have observed a high degree of sequence redundancy and dynamic equilibrium in the C-rich sequence of the c-MYC promoter. Several interesting points are noted from our study. First, in addition to the previous notion that loop isomers can be formed because not all cytosines are needed in the C^+^-C base pairs, our results show that loop isomers can also be formed by alternative base-pairing, as observed in the C11/20/23 sequence (Figure 4C). It is thus indicated that, unlike the G-quadruplex structure, the number of critical cytosines for the formation of I-motif is much reduced and the I-motif structure is intrinsically more flexible. Secondly, the I-motif formed in the c-Myc NHE III_1_ appears to be highly dynamic in its formation. The dynamic equilibrium between multiple structures caused by sequence and structure redundancy appear to provide entropy and stability to the overall I-motif structure. For example, the melting temperature of C11/20/23T is considerably lower than that of C11T and C20T (Figures 1C and Figure 2), thus the formation of the C23-C14 base pair, although less stable, and the potential presence of multiple conformers, appear to contribute significantly to the overall stability of the I-motifs formed in C11T and C20T. Moreover, we have observed two different dynamic processes between multiple conformers: a fast exchange equilibrium observed for the C11T and C20T sequences (Figures 4A and 4B), and a slow exchange equilibrium observed for the C11/20/23T sequence (Figure 4C). The fast exchange dynamic equilibrium appears to contribute more to the stability of the I-motif structure. While our study showed that in an oligonucleotide state the c-Myc I-motif forms at pH below 7 (e.g. pH 6.6), it has been recently shown that G-quadruplex and I-motif form at the two complementary DNA strands of the c-Myc promoter in a supercoiled plasmid under physiological pH and salt conditions at 37°C [27]. It is thus important to note that, with the dynamic equilibrium and the transcription-generated superhelicity, the I-motif secondary structures could form under physiological conditions.

The dynamic character appears to be intrinsic to the I-motif formed in the c-Myc promoter and could be important for both the potential formation of I-motif in vivo (by its stability) and its protein and ligand recognition (by its dynamic mixture). For example, the loop sizes and constitutions of an I-motif may be more important for protein or small molecule ligand recognition, while targeting individual I-motif structures may be less likely, especially considering the low diversity of the I-motif folding and molecular structures when compared to G-quadruplexes. It is noteworthy that the dynamic property can only be shown by using the non-chemically modified bases rather than using chemically modified bases needed for detailed NMR structure determination.

## Materials and Methods

### Sample preparation

The DNA oligonucleotides were synthesized as described previously [35], [44], [45], [46], [47], [48]. 6% ^15^N-labeled cytosine phosphoramidite was used for site-specific labeled DNA synthesis. The uniformly ^15^N-labeled cytosine phosphoramidite was purchased from Cambridge Isotope Laboratories. Unless otherwise stated, the NMR samples contained 0.5–1 mM DNA oligonucleotide. The pH of NMR samples was adjusted to 5.5 using trace amount of KOH or HCl, with no additional buffer added. The CD samples contained 10 µM DNA oligonucleotide in pH 5.5 10 mM potassium phosphate buffer.

*NMR experiments.* NMR experiments were performed on a Bruker DRX-600 spectrometer. Identifications of cytosine imino protons in site-specific labeled oligonucleotides were performed by one-dimensional ^15^N-filtered experiments, as described in our recent method paper [43]. The GE-JRSE HMQC [49], [50] were used to measure ^15^N-filtered spectra of imino protons in the hemiprotonated C^+^-C pairs. The ^15^N-^1^H transfer time was set to 12 ms, based on a series of 1D spectra with transfer time ranged from 4.5 ms to 16.6 ms. The relaxation delay of the ^15^N-filtered 1D spectrum was 1.5 s. The number of scans was set to 6k-12k. The carrier frequencies were set at the water peak in the ^1^H dimension and at 210 ppm in the ^15^N dimension.
